# Integrating Extended Reality Into Primary Care Chronic Pain Programs via the REDOCVR Intervention: Real-World Implementation Feasibility and Usability Study

**DOI:** 10.2196/82858

**Published:** 2025-10-31

**Authors:** Jose Ferrer Costa, Alexandra Fernandez Brusco, Christian Torrecillas Camacho, Elena Villabona Lopez, Melanie Rodriguez Belloso, Laura Villares Urgell, Pablo Serrano Barrena, Nuria Morán Bueno

**Affiliations:** 1 Research and Innovation Department, Badalona Serveis Assistencials, Pl Pau Casals 1, Badalona, 08911, Spain, 34 686512283; 2 Doctoral Programme in Health and Psychology, Universitat Oberta de Catalunya - UOC, Barcelona, Spain; 3 Research Support Unit, Metropolitana Nord, Jordi Gol i Gurina University Institute for Primary Health Care Research Foundation (IDIAPJGol), Mataró, Spain; 4 Multidisciplinary Research Group on Health and Society (GREMSAS) 2021 SGR 01484, Mataró, Spain; 5 Primary Care Services, Badalona Serveis Assistencials, Badalona, Spain

**Keywords:** augmented reality, chronic pain, deprescriptions, feasibility studies, patient-centered care, patient education as topic, primary health care, virtual reality

## Abstract

**Background:**

Chronic pain management in public health services often struggles with limited engagement, emotional burden, and medication use. Extended reality (XR) shows promise in specialized settings, but evidence for codesign and integration into primary care remains limited.

**Objective:**

This study aimed to examine the feasibility, usability, and real-world implementation of REDOCVR [RE (Reeducació), DOC (Dolor Crònic), VR (Virtual Reality)], an XR-supported psychoeducational program, and to explore preliminary clinical outcomes during its integration into chronic pain groups in public primary care centers.

**Methods:**

This was a nonrandomized, hybrid type 2 phased implementation study conducted in 3 primary care centers in Catalonia, Spain. The intervention built on existing multidisciplinary psychoeducational chronic pain groups led by psychologists and physiotherapists. In collaboration with patients, XR modules were codesigned and incorporated to enhance mindfulness, cognitive reframing, and motor activation activities already established in routine care. In total, 8 weekly sessions included 15‐20 minutes of this content, with a supervised medication tapering protocol included in later groups. Primary outcomes were implementation measures (adherence, tolerability, System Usability Scale, and satisfaction). Secondary outcomes included patient-reported clinical measures (Warwick-Edinburgh Mental Well-being Scale [WEMWBS], Hospital Anxiety and Depression Scale [HADS], Central Sensitization Inventory, and EuroQol – 5 Dimensions – 5 Levels) and medication changes, assessed at baseline, post-intervention, and 5-month follow-up.

**Results:**

In total, 42 participants were enrolled, and 36 (85.7%) completed the intervention and all assessments. Adherence was high, and no serious adverse events occurred, with minimal cybersickness reported (5.6%). Patient usability was strong (mean 81.4, 95% CI 75.6‐87.1), and overall satisfaction was high (mean 82.4, 95% CI 78.5‐86.4). Professional usability was moderate (mean 59.1, 95% CI 51.6‐66.5). Statistically significant improvements were observed in emotional well-being (Warwick-Edinburgh Mental Well-being Scale mean change 4.8, 95% CI 2.9‐6.7; Cohen *d*=0.86), anxiety (HADS-A –2.5, 95% CI –3.8 to –1.2; Cohen *d*=0.66), and depression (HADS-D –1.6, 95% CI –2.5 to –0.7; Cohen *d*=0.62) (all *P*<.001). Mobility improved significantly (median change –1.0, 95% CI –1.0 to 0.0, *P*=.02), while Pain/Discomfort showed a nonsignificant trend (*P*=.08). Among tapering participants (n=22), mean use of benzodiazepines decreased by 71.7% and opioids by 41.8% at 5 months.

**Conclusions:**

This study suggests that an XR-enhanced psychoeducational program can be incorporated into group-based chronic pain care within the public primary health care system. Exploratory improvements in emotional well-being, anxiety, depression, and reduced use of high-risk medications during supervised tapering indicate potential benefits, although causal inferences cannot be drawn given the feasibility design. These findings provide practical insights to inform refinement and progression to larger controlled studies evaluating scalability and long-term effects in routine primary care.

## Introduction

Chronic pain is common and disabling, with a recent systematic review estimating prevalence across European adults at roughly 21%, though individual studies range from 12% to 48% depending on criteria used [1]. In Spain, nationwide surveys report similar levels, with around 1 in 4 adults affected, and higher rates among women and in the 55‐75 age group [2]. Beyond prevalence, chronic pain is consistently linked with functional impairment, lower quality of life, and high health care use [1]. At a societal level, productivity losses from sick leave and early retirement have been estimated to consume up to 4% of GDP in some countries [3].

Primary care is where clinicians first diagnose, treat, and follow most people with chronic pain over time [4,5], heavily relying on pharmacological treatment. In this setting, providers frequently prescribe opioids, nonsteroidal anti-inflammatory drugs (NSAIDs), antidepressants, anticonvulsants, and benzodiazepines despite limited long-term benefit and safety concerns, particularly with opioid-benzodiazepine combinations [6,7]. Multidisciplinary group programs that combine physical, psychological, and self-management strategies may provide a safer and more comprehensive approach with effectiveness supported by evidence [8]. Yet, their accessibility remains inconsistent, constrained by structural and organizational barriers [9].

Extended reality (XR), including virtual reality (VR) and augmented reality (AR), is being explored as an addition to nonpharmacological pain strategies. Recent systematic reviews and early trials in specialist centers suggest XR interventions may help reduce pain intensity, support emotional regulation, and improve engagement [10-12]. A recent randomized trial reported that VR-based therapy improved clinical outcomes as well as brain imaging markers in chronic back pain [13]. Most of this evidence, however, comes from tightly controlled contexts rather than everyday practice, underscoring the need for feasibility and implementation studies in primary care and community settings [14,15].

We developed REDOCVR [RE (Reeducació), DOC (Dolor Crònic), VR (Virtual Reality)] in 2023 within a public primary care network in Catalonia, Spain, where multidisciplinary group programs for chronic pain were already established and delivered by psychologists and physiotherapists in line with the regional pathway [16]. Instead of developing a separate intervention or testing XR in a laboratory, we included it into existing sessions, keeping the same structure, timing, and professional roles. Clinicians and patients codesigned content, bringing together XR experiences for guided mindfulness, psychoeducation, distraction, and interactive motor exercises.

This paper examines the feasibility and implementation of REDOCVR in public primary care. By doing so, it directly addresses recent calls for research on how XR interventions can be integrated beyond specialist centers and sustained in routine clinical practice [14,15]. Its innovation lies in embedding XR into established multidisciplinary group programs, codesigned with patients and clinicians, rather than creating a new parallel service. This pragmatic approach ensured the program was conceived and delivered entirely within real-life clinical settings, focusing on implementation outcomes relevant for scalability.

## Methods

### Study Design and Setting

We conducted a hybrid type 2, nonrandomized, phased implementation study conducted in public primary care centers (PCC). The rollout was structured in three phases; this paper reports on Phases 1 and 2, which examined feasibility and optimization, while Phase 3 is ongoing and will be reported separately.

### Phases of Implementation

#### Phase 1: Codesign and Single-Site Feasibility

The first phase combined the participatory development process with a pilot group at PCC Apenins-Montigalà. Between September 2023 and February 2024, a core team of a psychologist, a physiotherapist, family physicians, a medical XR developer, technical partners, and patient representatives worked together to define content, delivery conditions, and safety procedures.

Professionals and patients cocreated the content to reflect the priorities of primary care, and immersive content was iteratively refined to ensure usability and acceptability. This included recording 360° videos with audio guides, reusing mindfulness modules from a previous project [17] (body scan and mindful breathing), and adapting the self-compassion module for the context of chronic pain. In parallel, AR hand-tracking exercises were designed in collaboration with professionals and patients to support safe, engaging movement, aligned with the routine interventions of the original program.

The pilot group (February-March 2024) completed eight 90-minute weekly sessions, with immersive content adjusted to the therapeutic goals of each session. Feasibility work at this stage focused on usability, acceptability, satisfaction, and iterative refinements of both content and technical procedures. XR was initially integrated into all weekly sessions in Phases 1 and 2; later adjustments are reported in the *Discussion* section.

#### Phase 2: Multisite Optimization

The program was expanded to include 2 additional PCCs while continuing at Apenins-Montigalà, comprising a total of 7 groups (3 at the original center, 2 at each new site). From the second group onward in each center, a supervised medication tapering protocol was introduced by family physicians, with scheduled reviews aligned to study timepoints.

#### Phase 3: Scale-Up

The program is currently extending to additional PCCs within Badalona Serveis Assistencials and to external institutions in the Catalan primary care system. This phase emphasizes readiness assessment, training of new professional teams, and preparation for replication beyond the initial network. The results from this phase will be reported separately.

### Participants and Eligibility Criteria

Eligibility was limited to adults enrolled in chronic pain group programs routinely offered at the PCCs. Participants had experienced chronic pain for at least 3 months, most often related to fibromyalgia, with no restrictions on pain site or intensity. Exclusion criteria, based on diagnoses documented in the medical record, included severe psychiatric or cognitive disorders, epilepsy, major neurological or sensory impairments, contraindications for physical activity, or barriers that prevented regular attendance at group sessions.

Recruitment was coordinated by the physiotherapist and psychologist leading the groups at each center. Patients invited to join the chronic pain groups were informed that the program included the option of using XR as part of our research study. They could choose to participate with or without the XR component and were free to continue in the group even if they later decided to withdraw from the study. In routine practice, referrals to the chronic pain groups were generally based on patients presenting features such as central sensitization, emotional distress, kinesiophobia, or limited response to prior treatments, although these factors were not applied as formal inclusion criteria for the study. At a dedicated evaluation visit, eligibility was confirmed by the physiotherapist or psychologist responsible for the group, and written informed consent was obtained together with baseline data. All patients who were invited chose to participate.

As the intervention was embedded in standard care, no randomization or blinding was applied. No formal sample size calculation was conducted, as these phases focused on feasibility and to inform the design of subsequent groups. The sample size was pragmatic, determined by patient enrollment in chronic pain groups during the study period. A minimum of 30 participants was considered sufficient to capture feasibility issues and guide refinements for broader implementation.

### Intervention

The intervention was delivered by psychologists, physiotherapists, and family physicians routinely leading group-based pain management programs. They received prior training and continuous technical support from the institutional innovation team, who had specific expertise in implementing XR in clinical care and codesigned the intervention content.

### Immersive Dose and Delivery Conditions

XR content was applied for 15‐20 minutes per session, with duration adjusted to the therapeutic focus and participants’ tolerance. All modules were delivered under direct facilitator supervision. Headsets (Meta Quest 2 and 3) were preloaded with the required content, sanitized between uses, and configured individually. Quest 3 devices were prioritized for AR passthrough sessions when available, given their superior visual quality.

To support acclimatization among first-time users, XR elements were introduced in a stepwise sequence. Early sessions began with a passive, audio-guided VR body scan, after which participants progressed to psychoeducational and mindfulness modules, and later to AR passthrough motor exercises and interactive tasks. This gradual progression reflected strategies already tested in other XR initiatives within our institution, where it proved effective in improving tolerability and reducing cybersickness [18].

### Final Intervention Components

**Figure 1.**
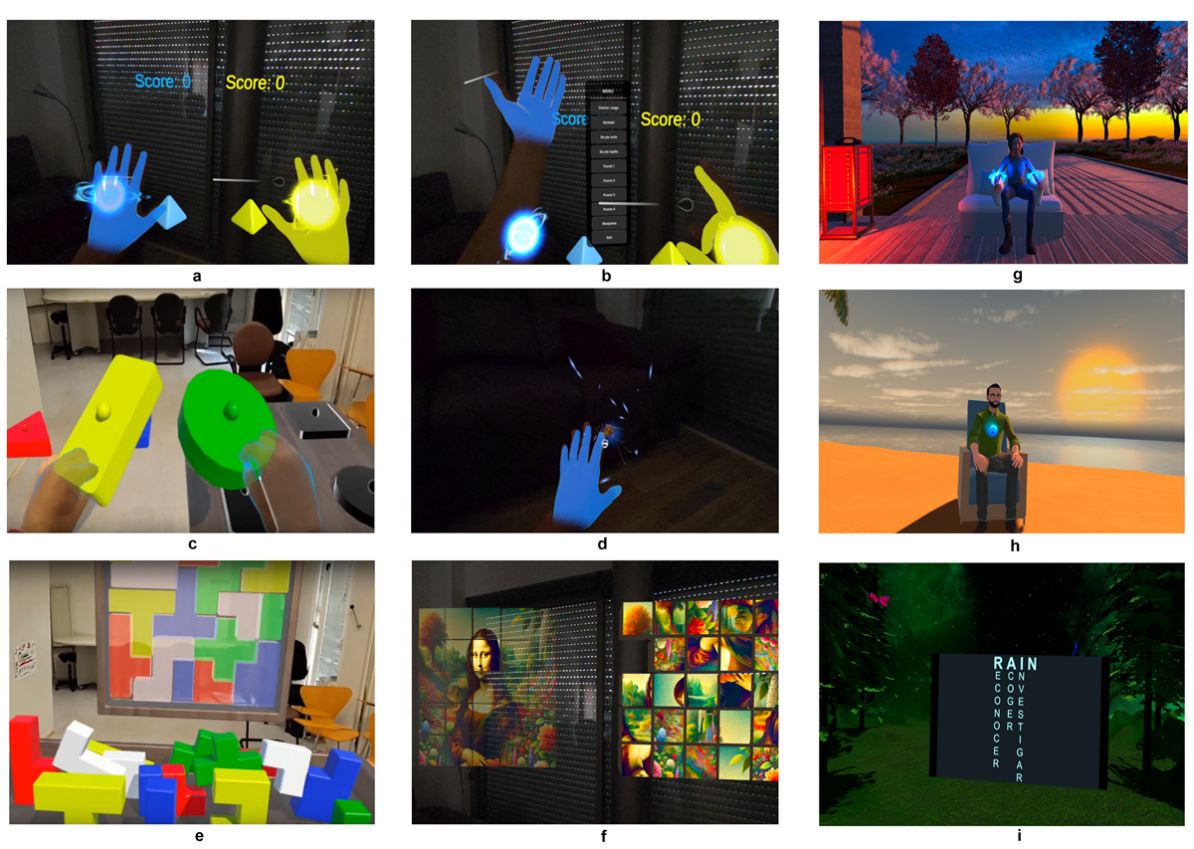
Immersive content developed and implemented in REDOCVR Phase 1: Panels a–f illustrate the REDOCVR augmented reality (AR) application created for guided physiotherapy sessions, designed to encourage safe movement and reduce kinesiophobia. Tasks were implemented in passthrough mode with hand tracking: (a) basic target interaction, (b) activation of the user interface menu by facing the left palm toward the user, (c) manipulation of 3D objects to promote reaching and coordination, (d) mosquito interception game to train postural control and attentional focus, (e) spatial reasoning puzzle based on Tetris blocks, and (f) visual memory puzzle with image fragments. Panels g–i show Unity-based VR environments from the *Projecte Benestar* program, integrated into group psychology sessions to support nonpharmacological pain management: (g) body scan practice in a zen garden environment, (h) guided mindful breathing exercise at a virtual beach, and (i) psychoeducational module introducing the RAIN (Recognize, Allow, Investigate, Nurture) framework for emotion regulation.

The codesign process resulted in 2 integrated components that became the stable structure of REDOCVR sessions (see Figures1  2). The XR content targeted 4 core domains relevant to chronic pain care in primary care: mindfulness and attentional focus; cognitive reframing and self-compassion; distraction for symptom regulation; and exercises to encourage safe, engaging movement.

**Figure 2.**
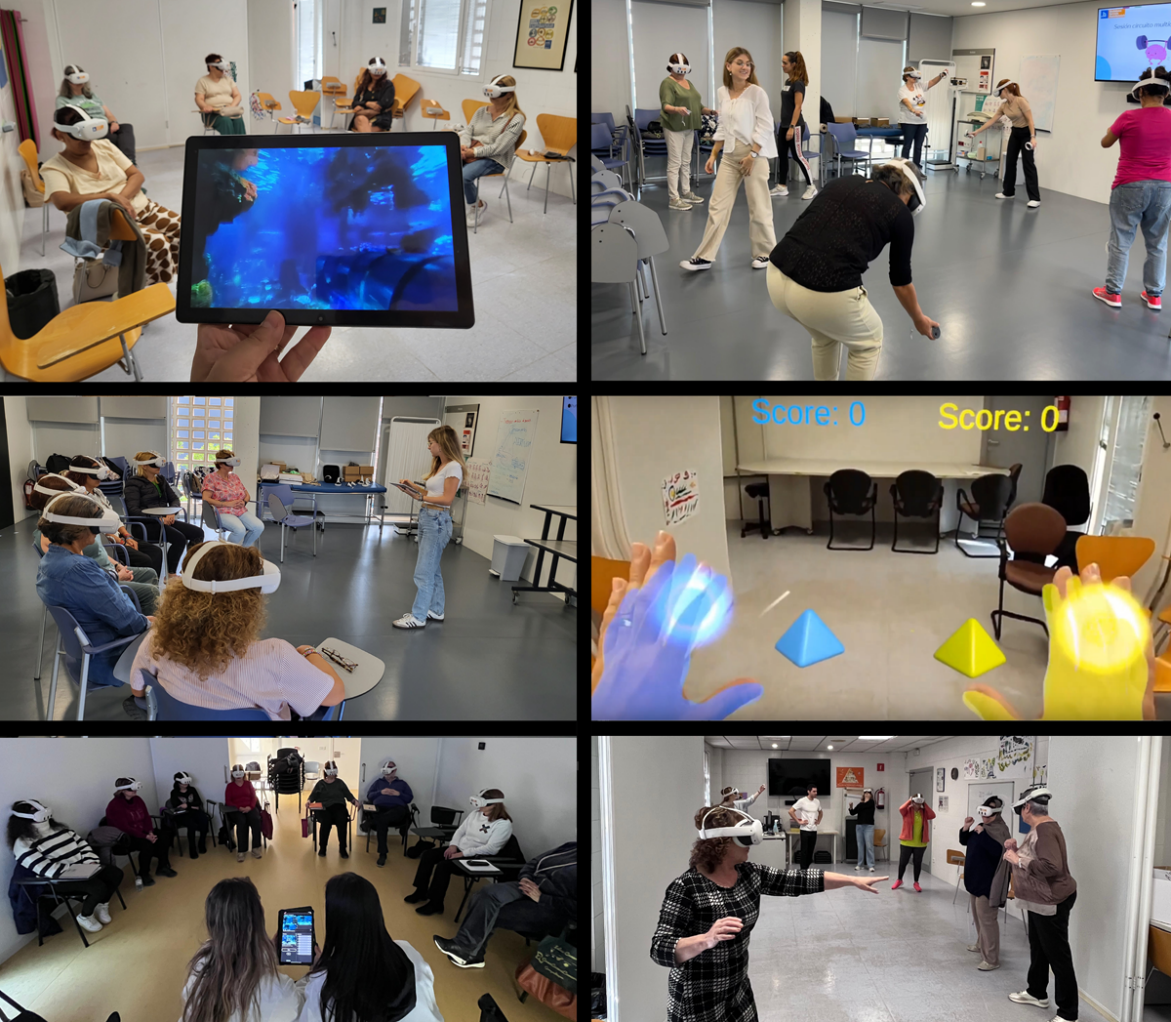
Implementation of XR content in REDOCVR sessions. The left column illustrates psychology-led sessions using the 360° virtual reality (VR) videos to support mindfulness and emotional awareness. A supervising tablet (top) allowed facilitators to monitor the video being displayed in the headsets, while group sessions (middle and bottom) were conducted with live guidance. The right column illustrates physiotherapy-led sessions. The top panel shows VR-based physical activity using controllers. The middle panel displays the augmented reality (AR) interface of the REDOCVR application with a gamified motor task from the participant’s perspective. The bottom panel depicts AR passthrough exercises using hand tracking during group movement practice. All sessions were supervised to ensure patient comfort and safety.

1. Psychology-focused sessions used VR modules to support psychoeducation, mindfulness, and emotional regulation. Depending on the session, participants worked with either the created 360° videos with audio guides (“Making peace with the pain” on natural beaches and a mindful dive in the Barcelona Aquarium) or Unity-based VR environments illustrating psychoeducational and cognitive reframing concepts. Modules were brief, noninteractive, and activated by simple hand-tracking. Together, they provided complementary approaches to emotional regulation, designed to be accessible to first-time XR users and flexible across sessions.
2. Physiotherapy-focused sessions integrated XR tools to encourage physical activation and body awareness. The REDOCVR AR app projected virtual objects into the physical space using passthrough and hand tracking, with tasks chosen by the physiotherapist according to functional goals: target interactions (touching floating objects), intercepting exercises requiring postural control (reacting to moving stimuli at 2 difficulty levels), and motor puzzles (shape-matching tasks). These modules incorporated elements of gamification, embodiment, and immersive presence to encourage motivation, although these factors were not directly assessed as outcomes. To provide a structured comparison, some sessions also used VR movement games from Immersive Oasis (a Spanish XR start-up). These games, operated with headset controllers rather than hand tracking, promoted functional movement while allowing evaluation of engagement and tolerability across different formats (AR vs VR, hand tracking vs controllers). Implementation fidelity was maintained by the standardized session scheme and calendar already established for the chronic pain groups, into which XR modules were integrated uniformly across sites.

All immersive content was available offline, preloaded in the headsets. Apps did not collect or transmit data; the only element requiring Wi-Fi was the optional Reality Telling tablet app for synchronizing playback of 360° videos.

### Outcome Measures

As this was an early-phase study, no explicit thresholds for feasibility or clinical outcomes were prespecified. The primary aim was to assess usability, tolerability, and feasibility of implementation under routine primary care conditions. The results were interpreted descriptively to guide adjustments in program delivery and inform the design of subsequent implementation phases, rather than to determine continuation or termination against predefined criteria.

#### Implementation Outcomes

Usability was assessed with 2 adaptations of the System Usability Scale (SUS), a validated instrument widely used in digital health research [19,20]. The patient version was reduced to 8 items, while the professional version retained the original 10 items with wording adapted to clinical use of XR. Both used 5-point Likert scales, with scores calculated following standard procedures and rescaled to a 0‐100 range. This approach aligns with recommendations to adapt usability tools for real-world XR implementations [21] and is supported by evidence that removing alternating positive-negative phrasing improves clarity without compromising validity [22-24].

Satisfaction and engagement were evaluated with a 10-item questionnaire developed for this study, covering perceived usefulness, ease of use, content quality, emotional impact, and physical comfort, scored on a 5-point Likert scale with responses similarly rescaled to a 0‐100 scale.

Adherence was defined as attendance at all scheduled sessions and completion of pre- or post-assessments. Most sessions were observed in person by the main investigator, with telephone debriefs where this was not possible. XR use and safety were monitored on a session-by-session basis, but without a structured or protocolized register; feedback was noted informally. Headset tolerability and safety were assessed with an 8-item questionnaire using 5-point Likert scales, covering comfort, fatigue, visual discomfort, dizziness, headache, and session interruption. Responses were analyzed descriptively at the item level rather than as a total score.

Qualitative feedback was obtained through a comments form, available at each session and again with the post-evaluation tests. The form contained a single free-text field without guiding questions. Responses were reviewed after each group to identify recurring perceptions. Informal remarks made during sessions were used to adjust delivery pragmatically but were not systematically examined.

#### Clinical Outcomes

Emotional well-being was measured with the 14-item Warwick-Edinburgh Mental Well-being Scale (WEMWBS), validated in Spanish samples with strong internal consistency (*α*=.90‐.93) [25,26].

Anxiety and depressive symptoms were assessed using the Hospital Anxiety and Depression Scale (HADS), Spanish version, which demonstrates strong internal consistency (*α*=.84 for anxiety, *α*=.85 for depression) and robust construct validity [27,28].

Central sensitization was evaluated with the Central Sensitization Inventory (CSI), scored 0‐100, with scores ≥40 indicating clinically relevant sensitization. The Spanish version shows solid psychometric properties in clinical populations [29,30].

Health-related quality of life was measured with the 5-level EuroQol (EuroQol – 5 Dimensions – 5 Levels [EQ-5D-5L]), using Spanish population norms to derive utility indices [31,32].

Medication use was reviewed in scheduled visits with family physicians, who checked electronic prescriptions together with patients and confirmed actual doses and any additional drugs not recorded. This measure was applied only in groups that included the deprescribing component.

### Data Management

#### Overview

We conducted assessments at baseline (month 0), post-intervention (month 2), and follow-up (month 5). Implementation outcomes were assessed at program completion, clinical outcomes at baseline and completion, and medication use at all 3 time points in groups where tapering was implemented. Questionnaires were administered on paper by the program facilitators, pseudonymized with unique alphanumeric codes, and transcribed into electronic spreadsheets. Data were stored on encrypted institutional servers with restricted access and regular backups, in compliance with Spanish data protection laws (Ley Orgánica de Protección de Datos y Garantía de Derechos Digitales 3/2018) and the European General Data Protection Regulation (EU 2016/679).

#### Statistical Analysis

Descriptive statistics were used to summarize sociodemographic data, clinical characteristics, and questionnaire responses. Continuous outcomes are presented as means with standard deviations or medians with interquartile ranges, according to distribution. Internal consistency was assessed for the adapted SUS questionnaires. Exploratory pre-post comparisons of clinical outcomes were conducted with paired-sample tests.

#### Reporting Guidelines

The study was reported in accordance with established guidelines. We followed the CONSORT extension for pilot and feasibility trials (see Checklist 1), the TIDieR checklist for intervention description (Checklist 2), RATE-XR for immersive technology research (Checklist 3), and iCHECK-DH for digital health implementation (Checklist 4).

### Ethical Considerations

Ethical approval was obtained from the Institut Universitari d’Investigació en Atenció Primària Jordi Gol Research Ethics Committee for both study protocols (refs. 24/047-P and 24/211-ACps), which are registered at ClinicalTrials.gov (NCT06361706 and NCT06964360). The REDOCVR XR content was implemented only as nontherapeutic support within a group psychoeducational program. After review by the Spanish Agency of Medicines and Medical Devices, it was formally classified as nonmedical device software, and no regulatory authorization was required.

All participants provided written informed consent before enrollment. Study data were deidentified prior to analysis, and no personal or health identifiers were collected by the XR software. Participants did not receive compensation for their participation. Figure 2 includes images where participants and clinicians are partially identifiable; written informed consent for publication of these images was obtained from all individuals depicted and archived by the research team.

## Results

### Sample Characteristics and Adherence

**Figure 3.**
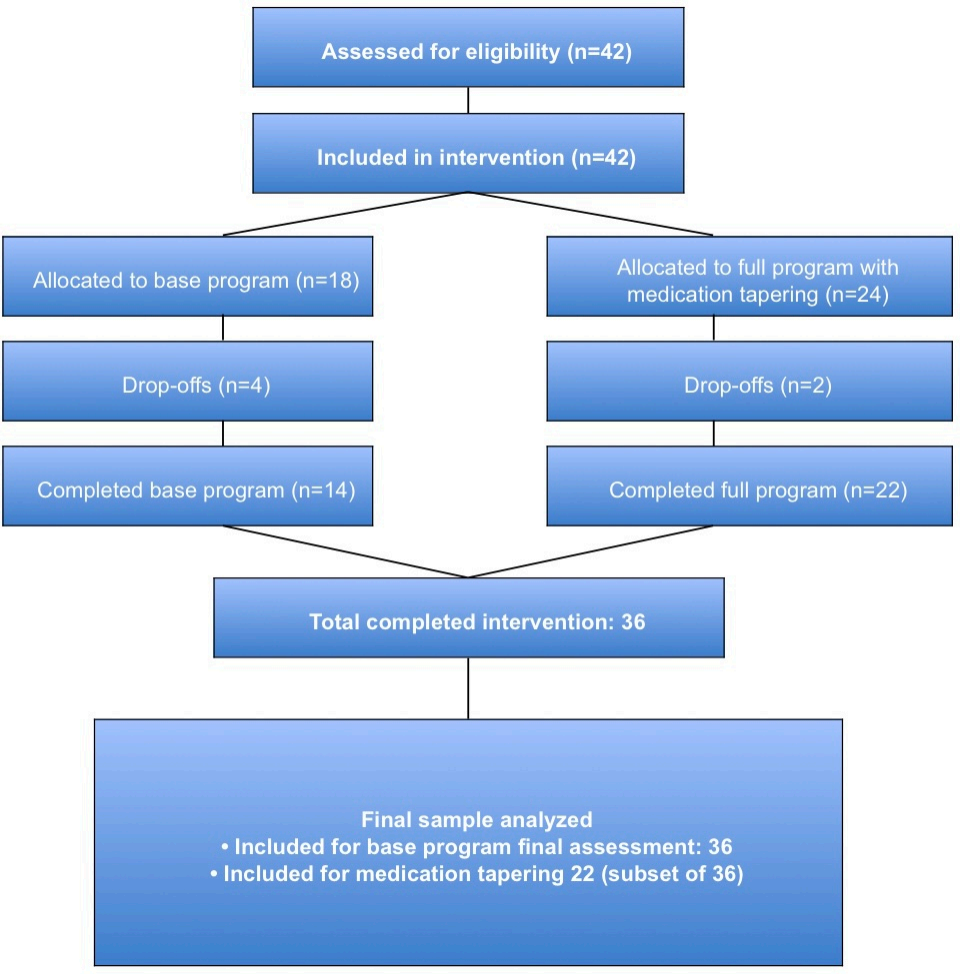
Flow of participants through the REDOCVR intervention groups. CONSORT-style diagram illustrating enrollment, dropout, and analysis for the 7 nonrandomized groups implemented in 2024. A total of 42 participants were enrolled across 3 primary care centers, of whom 36 (85.7%) completed the full intervention and both pre- and post-assessments. Analyses of clinical outcomes were based on these 36 participants. Medication-related outcomes were analyzed separately in the subset of 22 participants who took part in groups that included supervised medication tapering.

A total of 42 participants were enrolled across 7 intervention groups at 3 PCCs during 2024. Of these, 36 (85.7%) participants completed the full intervention and both pre- and post-assessments, with no missing outcome data among completers. Analyses of clinical outcomes were based on this final sample (n=36), with a mean age of 57.7 years (SD=9.36; range 40‐79) and a predominance of women (97.2%, n=35). Adherence was high, with the 6 dropouts attributed to patients’ logistical or scheduling conflicts, with no study-related reasons reported. Figure 3 illustrates a CONSORT-style participant flow diagram, while Table 1 details group composition across phases, from initial optimization to the later inclusion of supervised medication tapering.

**Table 1.** Distribution of REDOCVR intervention groups and participants completed in 2024^a^.

PCC^b^	Group	Phase	Implementation	Program sessions	Included (N=42), n	Completed post-evaluation (N=36), n	Medication tapering (N=22), n
Apenins-Montigalà	Group 1	Phase 1	Base program optimization	Feb-Mar 2024	6	4	-
Apenins-Montigalà	Group 2	Phase 2	Introduction of medication tapering	May-Jun 2024	6	6	6
Apenins-Montigalà	Group 3	Phase 2	Fully optimized program	Sept-Oct 2024	6	5	5
Progrés-Raval	Group 1	Phase 2	Base program only	Apr-May 2024	6	5	-
Progrés-Raval	Group 2	Phase 2	Fully optimized program	Sept-Nov 2024	6	6	6
Morera-Pomar	Group 1	Phase 2	Base program only	Apr-Jun 2024	6	5	-
Morera-Pomar	Group 2	Phase 2	Fully optimized program	Sept-Nov 2024	6	5	5

a Distribution of REDOCVR intervention groups completed in 2024 across 3 public primary care centers (PCCs) in Catalonia, Spain. The study followed a hybrid type 2, nonrandomized, phased implementation design. This paper reports on Phases 1 and 2, which focused on feasibility and optimization, while Phase 3 is ongoing and will be reported separately. The table shows participants’ details by implementation phase, session completion, and inclusion of supervised medication tapering where applicable.

b PCC: primary care center.

### Implementation Outcomes

#### System Usability

Patient-reported usability was high, with a mean SUS score of 81.4 (SD 17.1; 95% CI 75.6‐87.1). Most participants (83%, n=30) scored above the standard benchmark of 68, indicating strong perceived usability. Item-level results, including English translations of the adapted questionnaire items, are provided in Table 2. Internal consistency of the adapted 8-item scale was acceptable (Cronbach *α*=.70).

SUS scores from 8 health care professionals (3 psychologists, 3 physiotherapists, 1 family physician, and 1 biomedical technician) averaged 59.06 (SD 8.96; 95% CI 51.6‐66.5). Item-level ratings showed a mixed picture, with perceived contribution to patient care and clarity of simulation features scoring high, while system integration and need for support were rated lower. Internal consistency was low (Cronbach *α*=.28).

**Table 2.** Usability and satisfaction outcomes: patient and professional System Usability Scale (SUS), and patient satisfaction questionnaire.

Questions (English translation)	Mean (SD)	95% CI
(A) Patients—Adapted SUS (n=36, 8 items; Cronbach *α*=.70)^a^		
I think I would like to use this program frequently.	90.28 (22.58)	82.64‐97.92
I think the program is unnecessarily complex.	75.69 (34.06)	64.17‐87.22
I think the exercises are more engaging with the VR^b^ headset than conventional methods.	82.64 (32.08)	71.78‐93.49
I think the simulation options are clear and well integrated.	93.75 (19.25)	87.24‐100.26
I think some options are difficult to follow.	75.69 (34.58)	63.99‐87.4
I think people will learn to use this system easily.	81.25 (29.5)	71.27‐91.23
I felt comfortable using this system.	89.58 (21.86)	82.19‐96.98
I had to learn many things before being able to use the system.	61.81 (40.31)	48.17‐75.44
Total patients SUS score	81.35 (17.1)	75.56‐87.14
(B) Professionals—Adapted SUS (n=8, 10 items; Cronbach *α*=.28)^c^.		
I believe this program will help improve the quality of patient care.	84.38 (12.94)	73.56‐95.19
I believe the program interface is intuitive for the user.	75 (18.9)	59.2‐90.8
I believe the program can be easily integrated into daily clinical practice.	53.13 (36.44)	22.66‐83.59
I believe the simulation’s features are clear and relevant to patient care.	81.25 (11.57)	71.57‐90.93
Some features of this system may be difficult for patients to understand or use.	28.13 (20.86)	10.68‐45.57
I believe patients will be able to learn to use this system easily.	68.75 (22.16)	50.22‐87.28
I felt comfortable using this system for patient care.	78.13 (20.86)	60.68‐95.57
You needed additional time to learn how to use this system correctly.	40.63 (32.56)	13.4‐67.85
I believe the weight and design of the headset may be uncomfortable for some patients.	56.25 (34.72)	27.22‐85.28
I believe using the headset and controllers could be difficult for some patients.	25 (18.9)	9.2‐40.8
Total professionals SUS score	59.06 (8.96)	51.57‐66.55
(C) Patients—Satisfaction questionnaire (ad hoc, n=36, 10 items; Cronbach *α*=.81)^d^.		
Overall, how do you rate the VR experience during the chronic pain management sessions?	88.89 (13.94)	84.17‐93.61
What is your opinion on the usefulness of VR for pain reduction?	82.64 (15.61)	77.36‐87.92
Is the visual and image quality of the VR satisfactory for you?	88.19 (12.66)	83.91‐92.48
Is interaction with the VR during sessions easy to understand and use?	87.5 (17.42)	81.6‐93.4
How do you rate physical comfort while using VR?	80.56 (21.64)	73.23‐87.88
Has VR improved your concentration and ability to manage pain?	73.61 (21.5)	66.34‐80.89
What do you think about the variety of VR content available for your pain management sessions?	82.64 (18.73)	76.3‐88.98
How do you rate the support and guidance you received during the VR sessions?	92.36 (14.42)	87.48‐97.24
Is the duration of the VR sessions adequate for your goals?	75 (26.05)	66.19‐83.81
Do you intend to continue using VR in your pain management sessions?	72.92 (25.62)	64.25‐81.58
Totalpatients's satisfaction score	82.43 (11.69)	78.48‐86.39

a Patient System Usability Scale (SUS, adapted 8-item version; n=36). Total and item-level mean scores are presented on a 0–100 scale.

b VR: virtual reality.

c Professional SUS (standard 10-item version; n=8 health care professionals: 3 psychologists, 3 physiotherapists, 1 family physician, and 1 biomedical technician). Total and item-level mean scores are presented on a 0–100 scale.

d Patient satisfaction questionnaire (ad hoc; n=36). Items were rated on a 0–100 scale and are shown as mean (SD). All questionnaire items were translated into English for reporting.

#### Satisfaction

Overall satisfaction was high, with a mean score of 82.4 (SD 11.7; 95% CI 78.4‐86.4). The highest ratings were for support and guidance received (92.4), overall experience (88.9), and visual quality (88.2). Lower, though still positive, scores were reported for intention to continue use (72.9), session duration (75), and concentration and pain management (73.6). Item-level results with English translations are presented in Table 2.

#### Tolerability and Safety

Headset tolerability was generally high, with most participants disagreeing with statements indicating discomfort or adverse effects (Table 3). Dizziness (5.6%) and headache (5.6%) were rarely reported as frequent, and only 1 participant (2.8%) stopped a session due to discomfort. Fatigue in arms or hands was more common (33.3% agreement), likely reflecting the physical effort of interactive tasks rather than adverse effects. About half (50%) of participants rated the headset as comfortable for prolonged use. Overall, these findings suggest good tolerability with only occasional transient discomfort. Supervision confirmed correct use of XR without major incidents, and the open-ended feedback gathered during these sessions is described in the following section.

**Table 3.** Tolerability and safety of VR headsets (n=36)^a^.

Question (English translation)	Strongly disagree (%)	Disagree (%)	Neutral (%)	Agree (%)	Strongly agree (%)
The headset was very heavy and uncomfortable	50	8.3	8.3	25	8.3
Using the headset and controls was very complicated	61.1	13.9	8.3	11.1	5.6
I felt fatigue in my arms and fingers	44.4	13.9	8.3	25	8.3
I felt visual discomfort	72.2	11.1	2.8	11.1	2.8
I felt dizzy	63.9	22.2	8.3	2.8	2.8
I experienced headache	77.8	8.3	8.3	2.8	2.8
I had to stop the simulation due to discomfort	83.3	8.3	5.6	0	2.8
I think it would be comfortable to use these glasses for a long time	8.3	13.9	27.8	22.2	27.8

a Tolerability and safety of virtual reality (VR) headsets during REDOCVR sessions among patients with chronic pain in public primary care (n=36). Responses are reported as the percentage of participants selecting each option on a 5-point Likert scale (strongly disagree to strongly agree). Questionnaire items were translated into English for reporting.

#### Qualitative and Comparative Feedback

Patient feedback, collected through the comments form (see Multimedia Appendix 1 for thematic analysis), underscored both therapeutic and practical aspects of the program. The 360° mindfulness videos and the self-compassion module were consistently described as the most meaningful. Many participants noted that the immersive format of body scan and breathing practices made these techniques easier to follow, while psychologists emphasized in their debriefings that these elements were particularly useful for consolidating skills introduced in other sessions. A few participants reported brief emotional discomfort during emotion-focused modules; these reactions were anticipated as part of the process, addressed by clinicians, and did not disrupt participation.

Patients also drew clear contrasts between modalities. AR exercises were described as intuitive and less tiring, with passthrough providing orientation that supported group flow. VR was seen as more immersive and distracting, though some participants reported temporary dizziness, fatigue, or visual strain after longer interactions. Across groups, patients consistently preferred hand-tracking over controllers, citing its natural interaction and shorter setup time (see Multimedia Appendix 2 for a video of the program sessions and content).

Professionals echoed some of these observations and noted additional logistical challenges. VR sessions required extra time to configure safety areas and adjust headsets individually. At least 1 physiotherapist described difficulty leading a group of 8 patients by himself, as sequential headset setup left participants waiting; these issues were less frequent when 2 or 3 professionals co-led with technical support. Setting up the connection for tablet-controlled synchronization of 360° videos was also seen as an added burden, further highlighting the need for streamlined logistics (see Multimedia Appendix 2 for a video of the program sessions and content).

Across groups, patients suggested extending the program, increasing session frequency, and considering access to VR content outside the clinical setting.

### Clinical Outcomes

Normality assumptions determined the choice of test: paired-sample *t* tests were applied to normally distributed continuous outcomes (WEMWBS, HADS, CSI; Table 4), while Wilcoxon signed-rank tests were used for ordinal EQ-5D-5L domains (Table 5).

**Table 4.** Paired-sample *t* tests for normally distributed outcomes.

Variable	Mean difference (95% CI)	*P* value	Cohen *d*
Emotional wellbeing	4.83 (2.92 to 6.74)	<.001	0.86
HADS-Anxiety^a^	–2.47 (–3.75 to –1.20)	<.001	0.66
HADS-Depression	–1.64 (–2.53 to –0.75)	<.001	0.62
CSI^b^	–3.22 (–7.18 to 0.73)	0.1107	0.28

a HADS: Hospital Anxiety and Depression Scale.

b CSI: Central Sensitization Inventory.

**Table 5.** Wilcoxon signed-rank test results for EQ-5D-5L^a^ dimensions

EuroQol (EQ-5D-5L) dimensions	HL^b^ median change (95% CI)	*P* value	Effect size (r)	Interpretation
Mobility	–1.0 (–1.0 to 0.0)	0.0182	0.39	Significant improvement (moderate)
Self-care	0.0 (–0.5 to 0.0)	0.3374	0.16	No significant change (small)
Usual activities	0.0 (–0.5 to 0.5)	0.772	0.05	No change (very small)
Pain/Discomfort	–0.5 (–0.5 to 0.0)	0.084	0.29	Trend toward improvement (moderate)
Anxiety/Depression	0.0 (–0.5 to 0.0)	0.2869	0.18	No significant change (small)

a EQ-5D-5L: EuroQol 5-Dimension 5-Level questionnaire.

b HL: Hodges-Lehmann.

Table 6 presents pre-post values for clinical outcomes (n=36). Emotional well-being (WEMWBS) increased from 16.9 (SD 4.9) to 21.7 (SD 6.1), with a mean change of 4.8 (95% CI 2.9‐6.7, *P*<.001, Cohen *d*=0.86). Anxiety symptoms (HADS-Anxiety) decreased from 13.1 (SD 4.3) to 10.7 (SD 4.3), mean change –2.5 (95% CI –3.8 to –1.2, *P*<.001, Cohen *d*=0.66), and depressive symptoms (HADS-Depression) from 10.6 (SD 3.4) to 9 (SD 4.2), mean change –1.6 (95% CI –2.5 to –0.7, *P*<.001, Cohen *d*=0.62). Central sensitization (CSI) declined slightly, from 60.7 (SD 14.2) to 57.5 (SD 13.4), mean change –3.2 (95% CI –7.2 to 0.7, *P*=.11, Cohen *d*=0.28).

**Table 6.** Clinical outcomes: descriptive statistics and pre–post differences (n=36)^a^.

Variable	Mean (SD)	Median (IQR)	Range (Min-Max)	95% CI for mean
Descriptive statistics^b^				
Emotional wellbeing (Pre)	16.89 (4.92)	16.5 (6.25)	8‐26	15.22‐18.55
Emotional wellbeing (Post)	21.72 (6.14)	23.0 (8.25)	7‐34	19.64‐23.80
HADS-Anxiety (Pre)	13.14 (4.28)	13.5 (5.5)	4‐20	11.69‐14.59
HADS-Anxiety (Post)	10.67 (4.27)	10.5 (7.25)	4‐20	9.22‐12.11
HADS-Depression (Pre)	10.61 (3.43)	10.0 (5.0)	2‐18	9.45‐11.77
HADS-Depression (Post)	8.97 (4.23)	9.0 (5.0)	1‐21	7.54‐10.40
CSI^c^ (Pre)	60.72 (14.18)	60.0 (18.0)	34‐95	55.93‐65.52
CSI (Post)	57.50 (13.42)	58.0 (15.25)	33‐94	52.96‐62.04
Mobility (Pre)	2.19 (0.92)	2.0 (1.25)	1‐4	1.88‐2.51
Mobility (Post)	1.78 (0.83)	2.0 (1.0)	1‐4	1.50‐2.06
Self-Care (Pre)	1.72 (0.66)	2.0 (1.0)	1‐3	1.50‐1.95
Self-Care (Post)	1.58 (0.81)	1.0 (1.0)	1‐4	1.31‐1.86
Usual activities (Pre)	2.47 (1.00)	2.5 (1.0)	1‐5	2.13‐2.81
Usual activities (Post)	2.42 (1.20)	3.0 (2.0)	1‐5	2.01‐2.82
Pain/Discomfort (Pre)	3.69 (0.75)	4.0 (1.0)	2‐5	3.44‐3.95
Pain/Discomfort (Post)	3.44 (0.73)	4.0 (1.0)	2‐5	3.20‐3.69
Anxiety/Depression (Pre)	3.31 (1.14)	3.0 (1.0)	1‐5	2.92‐3.69
Anxiety/Depression (Post)	3.06 (1.33)	3.0 (2.0)	1‐5	2.61‐3.51

a Clinical outcomes for patients with chronic pain participating in the REDOCVR program in public primary care (n=36).

b Descriptive statistics for all outcome measures, including mean (SD), median (IQR), range, and 95% CI.

c CSI, Central Sensitization Inventory.

For quality of life (EQ-5D-5L), Mobility improved significantly (median change –1.0, 95% CI –1.0 to 0.0, *P*=.02, *r*=0.39), while Pain/Discomfort showed a nonsignificant trend toward improvement (median change –0.5, 95% CI –0.5 to 0.0, *P*=.08, *r*=0.29). Self-Care, Usual Activities, and Anxiety/Depression domains showed no significant changes.

#### Medication Tapering

Among the 22 participants who followed the deprescription protocol, medication use declined across several categories (Table 7). Between baseline and the 5-month review, the largest mean reductions were in benzodiazepines (71.7%, 6 complete discontinuations), opioids (41.8%, 5 discontinuations), and muscle relaxants (48%). More modest decreases were seen for NSAIDs and non-opioid analgesics. Patterns for antidepressants and pain modulators were mixed, with some patients starting new treatments during follow-up.

**Table 7.** Medication tapering by therapeutic category at 2 and 5 months post-intervention^a^.

Medication type	Timepoint (months)	Mean (%)	Median (%)	IQR (%)	Range (%)	N complete stops
NSAIDs^b^	2	29.86	33	45.75	0-100	1
NSAIDs	5	25.14	16.5	50	–100 to 100	2
Non-opioid analgesics	2	28.31	12.5	37.25	0-100	2
Non-opioid analgesics	5	30	12.5	49	0-100	2
Antidepressants	2	1.61	0	0	–100 to 100	3
Antidepressants	5	8.29	0	0	–100 to 100	6
Benzodiazepines	2	45	25	100	0-100	4
Benzodiazepines	5	71.7	100	45.75	0-100	6
Pain modulators	2	19.44	0	25	0-100	1
Pain modulators	5	34.22	25	50	0-100	2
Opioids	2	40.13	33	60	–100 to 100	5
Opioids	5	41.8	43	93	–100 to 100	5
Muscle relaxants	2	48	48	23	25-71	0
Muscle relaxants	5	48	48	23	25-71	0
Others	2	25	25	0	25-25	0
Others	5	100	100	0	100-100	1

a Medication tapering by therapeutic category at 2 and 5 months after participation in the REDOCVR program (n=22 patients with medication follow-up). Values represent percentage change in daily intake from baseline to each follow-up. Changes were calculated for each medication record and then aggregated by category. “N complete stops” indicates the number of participants who fully discontinued that medication. Negative values (eg, –100%) denote initiation of a medication not used at baseline. Data are reported as mean, median, interquartile range (IQR), and minimum-maximum percentage change for each category. “Others” refers to a single case of Versatis (lidocaine medicated plaster).

b NSAIDs: nonsteroidal anti-inflammatory drugs.

## Discussion

### Principal Findings

This study examined the early implementation of REDOCVR, an XR-enhanced group program for chronic pain in primary care. The findings suggest that immersive technologies can be integrated into existing care structures without disturbing routine delivery.

Satisfaction, tolerability, and usability indicators provide additional insight into how the program was received. Patients rated the sessions positively, reporting high satisfaction and minimal discomfort. Cybersickness was rare, and overall headset tolerance further supported the acceptability of XR in this population. The adapted 8-item SUS for patients reduced response burden while maintaining validity, consistent with prior analyses of SUS modifications [21-24,33]. In contrast, professional SUS scores showed low internal consistency, likely due to the small and heterogeneous sample. Such factors are known to reduce the stability of Cronbach *α* [34,35], whereas prior studies suggest that 10-item adaptations generally retain reliability [21,22]. Our findings, therefore, probably reflect contextual heterogeneity rather than flaws in the instrument itself.

An exploratory review of the open comments provided complementary insights into participants’ and professionals’ experiences with XR. Feedback suggests that XR was experienced less as a novel intervention in itself and more as a support that appeared to enhance engagement with the program’s therapeutic content. In psychology-led sessions, immersive tools supported mindfulness and self-compassion practices that some patients otherwise found difficult to understand. In physiotherapy-led sessions, AR passthrough helped participants perform movements in a safe and supervised way, while VR increased immersion and motivation, both encouraging patients to attempt physical activities they would normally avoid.

Exploratory clinical findings suggested improvements in emotional well-being, anxiety, and depression, with effect sizes in the moderate-to-large range. Statistically significant improvements were observed in Mobility on the EQ-5D-5L, while Pain/Discomfort showed only a nonsignificant positive trend. Among participants enrolled in the deprescription protocol, benzodiazepine, opioid, and NSAID intake decreased, and several participants achieved complete discontinuation. Although causality cannot be inferred, these results are consistent with the possibility that group-based interventions may complement structured deprescription strategies [36].

### Comparison With Prior Work

While our findings on patient usability and clinical outcomes align with the existing literature [10,37], the primary contribution of this study is its focus on pragmatic implementation within a public primary care setting. We have encountered barriers in early adoption similar to other reports related to technical setup and workflow adjustments [38]. Prior reviews have noted that most VR interventions for pain concentrate on supporting specific aspects of health literacy or self-management rather than integration into routine care [39]. By embedding XR within an established group program rather than running it in parallel, REDOCVR addresses adoption challenges noted in recent reviews [14,15,40] and shows how immersive tools can fit into everyday service delivery. Delivering XR in group sessions brings clear advantages, including peer support, efficiency, and easier integration into existing care pathways, though it comes at the expense of individualized tailoring. Meta-analytic evidence in chronic pain indicates that group-based approaches can achieve outcomes comparable to individual formats [41]. This appears particularly relevant in the context of the public health care system, where resource optimization is not only desirable but often decisive for adoption.

The improvements in emotional well-being and functional outcomes align with the psychoeducational and behavioral mechanisms targeted, consistent with results from mindfulness- and acceptance-based approaches [8,13]. The reduction in medication use also mirrors outcomes from other multidisciplinary primary care interventions that combine patient education with supervised tapering [5]. What our findings add is preliminary evidence that such effects may be achievable when immersive tools are embedded into routine group care, rather than tested only in parallel or experimental conditions.

### Limitations

The intervention was delivered to a modest number of groups within a supportive institutional setting, which may limit direct transferability to other contexts. Resources such as trained staff, headset availability, and organizational readiness facilitated implementation and should be considered when adapting the model elsewhere. Data collection on implementation fidelity was pragmatic. For instance, the dose of immersive exposure was guided by a time range (15‐20 min) and adjusted for tolerance, but not strictly logged for each individual patient in every session. Adherence was assessed through attendance records, while adverse effects were monitored through informal therapist feedback and a single post-intervention questionnaire, rather than a structured, session-by-session register. The qualitative component was restricted to an exploratory review of written comments. While this offered useful contextual insights for program refinement, it was not intended as a formal qualitative study.

Other limitations are typical of early-phase implementation research, and exploratory clinical outcomes should be interpreted with caution. The absence of randomization and a control group limits causal inference. Generalizability is constrained by the modest and predominantly female sample (97%), which reflects the higher prevalence of chronic pain among women and the predominance of female participation in primary care group interventions in Spain [2,42]. This imbalance may restrict the applicability of findings to male patients. Participants also represented a heterogeneous chronic pain population, and we did not stratify by phenotype (nociceptive, neuropathic, or nociplastic), which may have masked differential responses. The group delivery model, while resource-efficient and well-suited to public primary care, inevitably restricts opportunities for individual tailoring [40]. The use of self-reported measures introduces potential recall and social desirability bias. Positive findings may have been partly influenced by a novelty effect. Finally, the 5-month follow-up is insufficient to establish the durability of changes over time.

### Implications and Future Directions

Despite these constraints, the findings have already informed practical adjustments. Feedback from patients and professionals in Phases 1 and 2 reinforced the institutional support. Patients reported high satisfaction and usability, while professionals highlighted integration and workflow challenges. Combined with the clinical findings, these insights guided the Phase 3 refinements. The program was refined in collaboration with the full clinical team, leading to optimizations to improve feasibility.

In physiotherapy sessions, AR proved more suitable for larger groups, where supervision and interaction needed to be fluid, while VR was reserved for smaller groups. Both patients and professionals clearly favored hand-tracking, and all VR content was adapted accordingly, eliminating the need for controllers in the VR exercise games. Synchronizing 360° videos from the tablet required stable Wi-Fi, which was not always available. External routers were used to compensate, adding setup complexity. Efforts are underway to improve connectivity in PCCs and reduce facilitator workload.

Informed by early-phase findings and aligned with Catalan health system guidelines, the program will now follow the conventional format of 12 weekly sessions co-led by a psychologist and a physiotherapist, with selected sessions involving family physicians and nutritionists to reinforce healthy habits. XR use has been reorganized to fit within this structure, concentrated into 4 sessions—2 psychology-led (mindfulness and self-compassion) and 2 physiotherapy-led (motor activation). This targeted approach preserves therapeutic value while optimizing headset use and technical support across centers.

Future evaluation should focus on systematic data collection related to implementation fidelity. With XR use now organized into defined modules, session-by-session monitoring will allow more precise analysis of exposure, usability, and adverse effects across different modalities (eg, VR vs AR, passive vs interactive content). The expansion to additional centers and teams will be accompanied by structured pre-implementation focus groups and complementary usability strategies. Combined with established implementation frameworks, these steps are expected to generate the structured evidence needed to support broader adoption. By linking fidelity monitoring with pragmatic implementation, REDOCVR may help close the gap between immersive research and everyday chronic pain care.

### Conclusions

The first 2 phases of REDOCVR suggest that a person-centered, codesigned XR program can be incorporated in primary care chronic pain groups under real-world conditions. The intervention showed high adherence and favorable usability, and both patients and professionals valued its contribution to engagement and session delivery. Exploratory improvements in emotional well-being and reductions in high-risk medication use with supervised tapering are promising but should be interpreted with caution given the feasibility design.

This study is, to our knowledge, the first to develop and evaluate an XR program entirely within primary public health care. The findings offer practical guidance for refining XR-based interventions and preparing for wider scale-up. Ongoing work on Phase 3 across additional centers will further clarify long-term outcomes, scalability, and adaptability to different settings.

## Supplementary material

10.2196/82858 Multimedia Appendix 1 Thematic analysis of patient feedback with illustrative quotes.

10.2196/82858 Multimedia Appendix 2 REDOCVR BSA overview 2025: project content sample and real-life use in primary care.

10.2196/82858 Checklist 1 CONSORT Checklist (Feasibility Extension).

10.2196/82858 Checklist 2 TIDieR Checklist.

10.2196/82858 Checklist 3 RATE-XR Checklist.

10.2196/82858 Checklist 4 iCHECK-DH Checklist.

## Abbreviations

AR: augmented reality
CSI: Central Sensitization Inventory
EQ-5D-5L: EuroQol – 5 Dimensions – 5 Levels
HADS: Hospital Anxiety and Depression Scale
NSAIDs: nonsteroidal anti-inflammatory drugs
PCC: primary care center
REDOCVR: RE (Reeducació), DOC (Dolor Crònic), VR (Virtual Reality)
SUS: System Usability Scale
VR: virtual reality
WEMWBS: Warwick-Edinburgh Mental Well-being Scale
XR: extended reality
