# Supplementary material for: Integrating Extended Reality Into Primary Care Chronic Pain Programs via the REDOCVR Intervention: Real-World Implementation Feasibility and Usability Study
Source: JMIR XR Spat Comput. 2025 Oct 31;2:e82858. doi: 10.2196/82858 (PMC12671288; doi:10.2196/82858)
Supplement: Multimedia Appendix 1 [file xr-v2-e82858-s001.pdf]

Multimedia Appendix. Thematic analysis of patient feedback with illustrative quotes

Theme 1. Therapeutic benefits of the group programme

Feedback was collected through a comments form available during sessions and again with the post-evaluation tests at the end of each group. The form contained a single free-text field without guiding questions. Written responses were reviewed after the groups and organized thematically by the authors to identify recurring perceptions. This process was not part of the prespecified trial outcomes but provided complementary information for co-development and iterative optimization of the intervention.

Themes and subthemes are presented below with illustrative patient quotes (original language and English translation). Minor typographical errors in participant writing were preserved.

Sub-theme	Illustrative Patient Quote (Original)	English Translation
Positive experience and self-expression	“Me han gustado mucho estas sesiones, me he podido abrir y contar como esta mi vida y mi rutina, y sobretodo comentar como me siento.”	“I really enjoyed these sessions. I was able to open up and share my life and routine, and above all how I feel.”
Positive closure	“Muy gratificante, con pena de que termine.”	“Very rewarding, but sad to see it end.”
Group belonging	“Es satisfactoria en el sentido de que he conocido a gente como yo.”	“It is satisfying in the sense that I have met people like me.”
Cognitive understanding	“Era lo que yo necesitaba, saber lo que me pasaba, entendí que todo va en mi cerebro gracias al informe de médica.”	“It was what I needed, to know what was happening to me. I understood that everything is in my brain thanks to the doctor’s report.”

Theme 2. Safety and tolerability

Overall tolerance was good. A minority reported transient emotional or physical reactions that resolved without interrupting participation.

Sub-theme	Illustrative Patient Quote (Original)	English Translation
Mixed emotional responses	“He estado muy relajada pero a ratos he sentido ansiedad.”	“I was very relaxed, but I felt anxious at times.”
Triggered memories	“Al principio de la terapia hemos visto un video de relajación que me ha traído recuerdos de un familiar fallecido y he sentido ansiedad, con las gafas de visión 3D he conectado bien.”	“At the start we watched a relaxation video that brought back memories of a deceased relative and I felt anxious, though I connected well with the 3D glasses.”
Difficulty achieving relief	“Me cuesta mucho sentirme aliviado, cuando veo el video, tengo una sensación de vacio, ojalá logre encontrar esa tranquilidad algún momento.”	“It is hard for me to feel relieved when I watch the video. I feel a sense of emptiness. I hope I can find that calm at some point.”
Mild dizziness or fatigue	“Con la RV quizá me he mareado un poco, pero es mejor que la RA porque no me distraigo en lo que tengo alrededor.”	“With VR I may have felt a little dizzy, but it is better than AR because I do not get distracted by what is around me.”
Somatic reaction	“Me ha gustado, pero he notado que mi cuerpo temblaba mucho, no sé porque.”	“I liked it, but I noticed my body was shaking a lot, I do not know why.”

Theme 3. Practicality and usability

Patients compared modalities and interaction methods, and commented on concentration and session structure.

Sub-theme	Illustrative Patient Quote (Original)	English Translation
VR immersion reduces distraction	“Me gusta más que no se vea la sala, me distrae menos.” (RV)	“I prefer not seeing the room, it distracts me less.” (VR)
Alternative view with passthrough	“Me gusta más que se vea el escenario donde estás.” (RA)	“I like it more when you can see the setting you are in.” (AR)

Engagement and distraction from pain	"Genial la experiencia de las gafas, Jocs per distreure la ment i no sentir el DOLOR!!!"	"Great experience with the glasses, games to distract the mind and not feel the PAIN."
Hand-tracking preferred	"Prefiero que se utilicen las manos (libres) que los mandos."	"I prefer using hands rather than controllers."
Concentration, variable	"Experiencia buena, pero no he conseguido concentrarme en muchos momentos."	"Good experience, but I could not concentrate at many times."
Able to sustain focus	"La práctica… me ha gustado mucho, he podido seguirla sin que se me fueran los pensamientos a otra cosa, la he hecho con los ojos abiertos."	"I liked the practice a lot. I could follow it without my thoughts drifting, I did it with my eyes open."

Theme 4. XR modality comparisons for physical activation

Patients contrasted AR and VR during movement-based sessions.

Sub-theme	Illustrative Patient Quote (Original)	English Translation
VR more immersive and motivating	"Divertido. Prefiero las panorámicas (RV), el esfuerzo es llevadero."	"Fun. I prefer panoramic (VR), the effort is bearable."
XR helps movement	"Me ha gustado más la RV, es divertida y me ayuda, y los ejercicios… mueves el cuerpo."	"I liked VR more, it is fun and helps, and with the exercises you move your body."
AR acceptance with fatigue noted	"Me ha gustado más el de hoy (RA para ejercicio físico), pero también me he cansado mas."	"I liked today's session more (AR for physical activity), but I also felt more tired."

Theme 5. Suggestions for programme development

Patients asked for longer exposure, wider access, and complementary resources.

Sub-theme	Illustrative Patient Quote (Original)	English Translation
Longer duration and more sessions	"Únic punt que milloraria, allargar horari del taller i més sessions!! I fer realitat virtual en totes."	"The only point I would improve is to extend workshop hours and have more sessions, and do virtual reality in all of them."
Longer VR segments	"La sesión con RV se me ha hecho corta, pero muy amena."	"The VR session felt short, but very enjoyable."
More time overall	"Sugerencia, mayor duración de la terapia en el tiempo, no solo 8 semanas."	"Suggestion, greater duration of the therapy over time, not only 8 weeks."
Broader access and referral	"Más al alcance de la población. Prevención de recaído, quiero que se quede (el programa)."	"More within reach of the population. Relapse prevention, I want it to stay (the programme)."
Awareness and referral pathways	"No me enteria de que esto existia si mi dra no me hubiese comentado."	"I would not have known this existed if my doctor had not told me."
Home access to XR	"Me gustaría tenerlas (las gafas)."	"I would like to have the glasses."
Access to audio materials	"Estaria bien que se nos facilitaran los audios de las meditaciones."	"It would be good if we were given the meditation audios."
Multidisciplinary input	"Jo he trobado a faltar un nutricionista."	"I missed having a nutritionist."

Notes

Languages are as written by participants, Spanish or Catalan. Minor typographical errors preserved.

One therapist note documented "4 de 5 prefieren la RV a la RA, todas prefieren sin mandos" in Progrés Grup 1, which triangulates patient preferences without adding new outcomes.
