# Supplementary material for: Integrating Extended Reality Into Primary Care Chronic Pain Programs via the REDOCVR Intervention: Real-World Implementation Feasibility and Usability Study
Source: JMIR XR Spat Comput. 2025 Oct 31;2:e82858. doi: 10.2196/82858 (PMC12671288; doi:10.2196/82858)
Supplement: Checklist 1 [file xr-v2-e82858-s003.pdf]

CONSORT Extension for Pilot and Feasibility Studies Checklist for 'Integrating Extended Reality Into Primary Care Chronic Pain Programs: Real-World Feasibility Study of the REDOCVR Intervention' (Ferrer Costa et al, 2025)

Item #	Section / Topic	Pilot/Feasibility Item Description	Verification	Manuscript Location
1a	Title & Abstract	Identification as a pilot or feasibility randomized trial in the title	Fully: Title includes "Feasibility Study," clearly indicating its early-phase nature.	Title
1b	Title & Abstract	Structured summary of pilot trial design, methods, results, and conclusions (per CONSORT for abstracts)	Fully: Abstract follows the required structured format with clear reporting.	Abstract
2a	Introduction – Background	Scientific background and explanation of rationale for future definitive trial, and reasons for the pilot trial	Fully.	Introduction
2b	Introduction – Objectives	Specific objectives or research questions for the pilot trial	Fully.	Introduction
3a	Methods – Trial design	Description of pilot trial design (e.g., parallel, factorial) including allocation ratio, and whether changes to methods were made after pilot start, with reasons	Fully: Nonrandomised, phased.	Methods
3b	Methods – Changes to trial design	Important changes to design or methods after trial commencement, with reasons	Fully.	Methods
4a	Methods – Participants	Eligibility criteria for participants	Fully.	Methods
4b	Methods – Setting & locations	Settings and locations where the data were collected	Fully.	Methods
4c	Methods – Identification	How participants were identified and consented	Fully: Invited via care.	Methods
5	Methods – Interventions	Detailed description of the interventions intended for each group and how and when they were actually administered	Fully.	Methods
6a	Methods – Outcomes	Completely defined prespecified assessments or measurements to address each pilot trial objective, including how and when they were assessed	Fully: Prespecified.	Methods
6b	Methods – Changes to outcomes	Any changes to pilot trial assessments or measurements after the pilot trial commenced, with reasons	None noted.	N/A
6c	Methods – Progression criteria	Pre-specified criteria used to judge whether or how to proceed with a future definitive trial	Fully.	Methdos
7a	Methods – Sample size	Rationale for numbers in the pilot trial (not a formal sample size calculation for efficacy)	Fully: No formal calc.	Methods
7b	Methods – Interim analyses	When applicable, explanation of any interim analyses and stopping guidelines	N/A: Non-randomised feasibility design, so randomisation/blinding not applicable.	Methods
8a	Methods – Randomisation sequence generation	Method used to generate the random allocation sequence	N/A: Non-randomised feasibility design, so randomisation/blinding not applicable.	Methods
8b	Methods – Randomisation type & restrictions	Type of randomisation; details of any restriction (e.g., blocking, stratification)	N/A (and compliant): Same as above; no randomization means no type/restrictions to report.	Methods

9	Methods – Allocation concealment	Mechanism used to implement the random allocation sequence, describing any steps to conceal the sequence until interventions were assigned	N/A (and compliant): Not relevant without randomization.	Methods
10	Methods – Implementation	Who generated the random allocation sequence, enrolled participants, and assigned participants to interventions	N/A (and compliant): No sequence generation or assignment process to describe.	Methods
11a	Methods – Blinding	Whether participants, care providers, and outcome assessors were blinded to group assignment, and how	N/A: Non-randomised feasibility design, so randomisation/blinding not applicable.	Methods
11b	Methods – Similarity of interventions	If relevant, description of the similarity of interventions	N/A (and compliant): No comparator group, so similarity not applicable.	Methods
12	Methods – Statistical methods	Statistical methods used to address each pilot trial objective and, where applicable, methods for additional analyses	Fully: Descriptive.	Methods
13a	Results – Participant flow	Numbers of participants who were screened, eligible, randomised, received intended treatment, and were analysed for the primary outcome	Fully: Figure 2 provides a clear participant flow diagram as recommended.	Results (Sample characteristics and adherence); Fig. 2
13b	Results – Losses & exclusions	Losses and exclusions after randomisation, together with reasons	Fully addressed: Drop-offs explicitly reported (total n=6: Base=4, Full=2), with reasons (mainly logistical or scheduling conflicts). No post-inclusion exclusions mentioned.	Results (Sample characteristics and adherence); Fig. 2
14a	Results – Recruitment	Dates defining the periods of recruitment and follow-up	Fully addressed: Recruitment periods (during 2024, across seven groups/phases at three centers), with flow-up implied through completion and follow-up assessments (e.g., post-intervention at month 2, follow-up at month 5).	Results (Sample characteristics and adherence); Methods (Phases)
14b	Results – Trial stopped early	Why the pilot trial ended or was stopped	N/A (and compliant): The study completed all planned phases (1-2 reported, 3 ongoing as intended); no early termination or stopping reasons mentioned.	Results (no indication of stop); Methods (Design – phased, ongoing)
15	Results – Baseline data	Baseline demographic and clinical characteristics of each group	Fully.	Results
16	Results – Numbers analysed	Number of participants (denominator) included in each analysis, and whether the analysis was by original assigned groups	Fully.	Results
17	Results – Outcomes & estimation	For each objective, results for each group, and the estimated effect size and its precision (e.g., 95% CI)	Fully.	Results
18	Results – Ancillary analyses	Results of any other analyses performed that could be used to inform the future definitive trial	Fully.	Results
19	Results – Harms	All important harms or unintended effects in each group	Fully.	Results
20	Discussion – Limitations	Pilot trial limitations, addressing sources of potential bias and uncertainty, and imprecision of results	Fully.	Discussion
21	Discussion – Generalisability	Generalisability (external validity) of pilot trial methods and findings to future definitive trial and other settings	Fully.	Discussion
22	Discussion – Interpretation	Interpretation consistent with pilot trial objectives and findings, balancing potential benefits and harms, and considering other relevant evidence	Fully.	Discussion
23	Other – Registration	Registration number and name of trial registry	Fully.	Methods

24	Other – Protocol	Where the pilot trial protocol can be accessed, if available	Fully.	Abstract, Methods
25	Other – Funding	Sources of funding and other support, role of funders	Fully.	Funding
26	Other – Ethical approval	Name of ethics committee and reference number	Fully.	Methods

Adapted from: Eldridge SM, Chan CL, Campbell MJ, et al. CONSORT 2010 statement: extension to randomised pilot and feasibility trials. BMJ. 2016;355:i5239. doi:10.1136/bmj.i5239
