# Supplementary material for: Integrating Extended Reality Into Primary Care Chronic Pain Programs via the REDOCVR Intervention: Real-World Implementation Feasibility and Usability Study
Source: JMIR XR Spat Comput. 2025 Oct 31;2:e82858. doi: 10.2196/82858 (PMC12671288; doi:10.2196/82858)
Supplement: Checklist 2 [file xr-v2-e82858-s004.pdf]

Template for Intervention Description and Replication (TIDieR) checklist for 'Integrating Extended Reality Into Primary Care Chronic Pain Programs: Real-World Feasibility Study of the REDOCVR Intervention' (Ferrer Costa et al, 2025)

Item #	Item	Explanation	Verification	Manuscript Location
1	Brief name	Provide the name or a phrase that describes the intervention.	Fully: The intervention is consistently and clearly named "REDOCVR".	Title, Abstract, Introduction
2	Why	Describe any rationale, theory, or goal of the elements essential to the intervention.	Fully: The introduction clearly explains the rationale for using XR to enhance non-pharmacological pain strategies in primary care.	Introduction
3	What (materials)	Describe any physical or informational materials used in the intervention, including those provided to participants or used in intervention delivery or training. Provide information on where these can be accessed.	Partially: Details hardware (Meta Quest 2/3) and software sources (Projecte Benestar, Immersive Oasis, in-house). Does not provide direct URLs or access links (in-house product not available commercially)	Methods (Co-Design and Development)
4	What (procedures)	Describe each of the procedures, activities, and/or processes used in the intervention, including any enabling or support activities.	Fully: Details the procedures for psychology-led (VR mindfulness) and physiotherapy-led (AR motor exercises) sessions, including the acclimatization process.	Methods (Final intervention components)
5	Who provided	For each category of intervention provider (e.g., psychologist, physiotherapist), describe their expertise, background, and any specific training given.	Partially: States delivery by trained psychologists, physiotherapists, and physicians. Mentions "prior training and continuous technical support" but does not detail the training content or duration.	Methods (Intervention Description)
6	How	Describe the modes of delivery (e.g., face-to-face, online) of the intervention and whether it was provided individually or in a group.	Fully: Clearly states the intervention was delivered in face-to-face group sessions.	Methods (Design and Setting)
7	Where	Describe the type(s) of location(s) where the intervention occurred, including any necessary infrastructure or relevant features.	Fully: Specifies the location as three public primary care centers in Catalonia, Spain.	Methods (Design and Setting)
8	When and how much	Describe the number of times the intervention was delivered and over what period of time, including the number of sessions, their schedule, and their duration, intensity, or dose.	Fully: Specifies the "dose" as 8 weekly sessions with 15–20 minutes of immersive content per session.	Methods (Immersive dose and delivery)
9	Tailoring	If the intervention was planned to be personalised, titrated, or adapted, describe what, why, when, and how.	Fully: Describes how content was chosen based on therapeutic goals and duration was adjusted based on participant tolerance.	Methods (Immersive dose and delivery)
10	Modifications	If the intervention was modified during the course of the study, describe the changes (what, why, when, and how).	Fully: Clearly reports iterative refinements, such as abandoning the tablet synchronization and standardizing session content.	Discussion (Implications and Future Directions)
11	How well (planned)	If intervention adherence or fidelity was assessed, describe how and by whom, and if any strategies were used to maintain or improve fidelity.	Fully: Transparently states that sessions were supervised but "no structured fidelity checklist or protocol was applied."	Methods (Implementation outcomes)
12	How well (actual)	If intervention adherence or fidelity was assessed, describe the extent to which the intervention was delivered as planned.	Partially: Reports high adherence (85.7% attendance) as a proxy for fidelity. Does not formally assess the extent to which content was delivered as planned.	Results (Sample characteristics and adherence)

Adapted from: Hoffmann TC, Glasziou PP, Boutron I, et al. Better reporting of interventions: template for intervention description and replication (TIDieR) checklist and guide. BMJ. 2014;348:g1687. doi:10.1136/bmj.g1687
