# Supplementary material for: Integrating Extended Reality Into Primary Care Chronic Pain Programs via the REDOCVR Intervention: Real-World Implementation Feasibility and Usability Study
Source: JMIR XR Spat Comput. 2025 Oct 31;2:e82858. doi: 10.2196/82858 (PMC12671288; doi:10.2196/82858)
Supplement: Checklist 3 [file xr-v2-e82858-s005.pdf]

RATE-XR (reporting for the early-phase clinical evaluation of applications using extended reality) checklist for 'Integrating Extended Reality Into Primary Care Chronic Pain Programs: Real-World Feasibility Study of the REDOCVR Intervention' (Ferrer Costa et al, 2025)

Theme	Item #	Summarized Recommendation	Verification	Manuscript Location
**Title and abstract**				
Title	1	Identify the study as an early clinical evaluation of an XR application in the title.	Fully: Title clearly includes "Real-World Feasibility Study" and "Extended Reality."	Title
Abstract	I	Provide a structured abstract summarizing the study's rationale, methods, results, and conclusions.	Fully: A structured abstract is provided with all required components.	Abstract
**Introduction**				
Clinical problem and existing evidence	2	Describe the clinical problem and the existing evidence or standard treatments.	Fully: The introduction details the challenges of chronic pain management and the evidence gap for XR in primary care.	Introduction
Introduction of the application	3	Introduce the XR application, its hypothesized effect, and any previous research.	Fully: The REDOCVR program is introduced, along with its rationale and connection to prior work.	Introduction
Objectives	II	Specify the study's objectives or hypotheses.	Fully: The objective to evaluate feasibility, usability, and preliminary outcomes is clearly stated.	Abstract (Objective)
**Methods and analysis**				
Trial design and reporting	III	Provide reference to ethical approval, study protocol, and public registration.	Fully: Provides ethics committee references and two ClinicalTrials.gov registration numbers.	Methods (Ethics)
Trial design and reporting	IV	Describe the study design and rationale, using a flow diagram if helpful.	Fully: The hybrid type 2, non-randomized design is described. A participant flow diagram (Fig 2) is included.	Methods (Design), Results (Fig 2)
Participants and setting	4	Describe the setting and locations where data was collected and the XR app was used.	Fully: Specifies the setting as three public primary care centers in Catalonia, Spain.	Methods (Design and Setting)
Participants and setting	5a	Describe participant selection, recruitment, and eligibility criteria.	Fully: Details the recruitment process and provides clear inclusion/exclusion criteria.	Methods (Participants)
Participants and setting	5b	Describe who delivered the XR application and the training they received.	Partially: States delivery by trained psychologists, physiotherapists, and physicians with technical support, but does not detail the specific content or duration of the training.	Methods (Intervention Description)
Intervention and procedures	6	Describe the XR application's content, hardware, protocol, and setup.	Fully: Details hardware (Quest 2/3), software content (VR/AR modules), and setup procedures.	Methods (Co-Design, Intervention)
Intervention and procedures	7	Describe the development process of the XR application.	Fully: Describes the co-design process involving clinicians, developers, and patients.	Methods (Co-Design and Development)

Intervention and procedures	8	Describe the participant timeline, including all procedures and assessments.	Fully: The timeline (baseline, post-intervention at 2 months, follow-up at 5 months) is clearly described.	Methods (Data Management)
Intervention and procedures	V	Describe the control condition or provide a rationale for its absence.	Fully: As a single-arm feasibility study, the manuscript correctly notes the absence of a control group and addresses this in the limitations.	Discussion (Limitations)
Outcomes	VI	Describe all prespecified primary and secondary outcomes.	Fully: All implementation and clinical outcomes are clearly defined and listed.	Methods (Outcome Measures)
Outcomes	9	Describe how safety, harm, and other XR-specific outcomes were assessed.	Fully: Details the use of a tolerability questionnaire for safety/harm and the SUS for usability.	Methods (Outcome Measures)
Sample size	VII	Provide a justification for the sample size.	Fully: Correctly states that no formal sample size calculation was performed, as is appropriate for a feasibility study.	Methods (Participants)
Analysis	VIII	Describe the statistical methods used to analyze outcomes.	Fully: The statistical analysis plan is clearly described.	Methods (Statistical Analysis)
Protocol alterations	IX	Describe any changes made to the protocol after the study began.	Fully: Describes iterative refinements, such as adding the tapering protocol and abandoning the tablet sync.	Methods (Co-Design), Discussion
**Results**				
Participant flow and recruitment	X	Report the recruitment timeframe and participant flow using a diagram.	Fully: The timeframe (during 2024) is stated, and participant flow is detailed in Figure 2.	Results (Sample characteristics)
Baseline data	XI	Provide baseline demographic and clinical characteristics.	Fully: Baseline characteristics are provided in the text and in Table 4.	Results (Sample characteristics, Table 4)
Main results	XII	Report results for all prespecified outcomes.	Fully: All outcomes are reported in the text and detailed in Tables 2, 3, 4, and 5.	Results
XR and human factors	10	Report on XR usage data (e.g., duration, frequency, interruptions).	Fully: Reports duration (15-20 min/session), frequency (weekly for 8 weeks), and interruptions (one participant).	Methods (Immersive dose), Results (Tolerability)
XR and human factors	11	Report results for assessed XR-specific outcomes (e.g., usability, presence).	Partially: Reports extensively on usability (SUS) and satisfaction. Does not report on other XR outcomes like presence or embodiment using formal scales.	Results (System usability, Satisfaction)
Safety and harms	12	Report on all safety and harm outcomes, including unintended effects.	Fully: Tolerability and safety are detailed in the text and Table 3, with no serious adverse events noted.	Results (Tolerability and safety)
**Discussion and conclusion**				
Generalizability and impact	13	Discuss the study's impact, generalizability, and implementation barriers.	Fully: The discussion covers the potential impact, limitations to generalizability, and implementation challenges (e.g., professional setup burden).	Discussion

Safety and harms	14	Discuss safety and harm findings and their implications for future use.	Fully: Discusses the good safety profile and notes that transient discomfort is manageable with supervision.	Discussion (Principal Findings)
Ethics	15	Describe ethical considerations, including benefits and risks.	Fully: Ethical approval and regulatory considerations are detailed in the methods section.	Methods (Ethics)
Strengths and limitations	XIII	Discuss study strengths and limitations, including potential sources of bias.	Fully: A dedicated section thoroughly discusses the study's limitations.	Discussion (Limitations)
Conclusion	16	Provide a conclusion that accurately interprets the findings.	Fully: The conclusion accurately summarizes the feasibility findings and suggests future directions.	Conclusions
**Statements**				
Funding and conflicts of interest	XIV	Disclose all funding sources and any potential conflicts of interest.	Fully: Funding sources and a conflicts of interest statement are both provided.	Funding, Conflicts of Interest
Application	17	Indicate the application's commercial status, availability, and regulatory compliance.	Fully: States the AEMPS classification (non-medical device software) and that content was co-designed or from third parties.	Methods (Ethics, Co-Design)

Note: XR-specific items are numbered in Arabic numerals; generic items are numbered in Roman numerals.

Adapted from: Vlake JH, Drop DLQ, Van Bommel J, et al. Reporting Guidelines for the Early-Phase Clinical Evaluation of Applications Using Extended Reality: RATE-XR Qualitative Study Guideline. J Med Internet Res. 2024;26:e56790. doi:10.2196/56790
