# Supplementary material for: Integrating Extended Reality Into Primary Care Chronic Pain Programs via the REDOCVR Intervention: Real-World Implementation Feasibility and Usability Study
Source: JMIR XR Spat Comput. 2025 Oct 31;2:e82858. doi: 10.2196/82858 (PMC12671288; doi:10.2196/82858)
Supplement: Checklist 4 [file xr-v2-e82858-s006.pdf]

**iCHECK-DH: Guidelines and Checklist for the Reporting on Digital Health Implementations for 'Integrating Extended Reality Into Primary Care Chronic Pain Programs: Real-World Feasibility Study of the REDOCVR Intervention' (Ferrer Costa et al, 2025)**

| Item # | Section | Item | Mandatory (M) / Non-Mandatory (NM) | Description | Verification | Manuscript Location |
|---|---|---|---|---|---|---|
| 1 | Title | Title | M | Identification as an implementation report, and description of the implementation in the title and/or keywords | Fully: Title includes "Real-World Feasibility Study," clearly identifying it as an implementation report. | Title |
| 2 | Abstract | Abstract | M | Provide a summary of the key elements of the implementation report, including description of the strategy, intervention, key elements, health outcomes, and KPIs | Fully: The structured abstract details the implementation strategy (embedding in existing care), the intervention, and key feasibility outcomes. | Abstract |
| 3 | Introduction | Context | M | Describe geographical areas, organizations, target populations, and context; consider barriers/facilitators; state alignment with national strategy; describe stage of implementation | Fully: Describes the geographical context (Catalonia), organizations (public PCCs), and alignment with the national health strategy. | Introduction, Discussion |
| 4 | Introduction | Problem statement | M | Describe the problem, challenge, or deficiency addressed, referencing WHO digital health classification if applicable | Fully: Clearly defines the clinical problem of chronic pain management and the evidence gap for XR implementation. | Introduction |
| 5 | Introduction | Similar interventions | M | Mention if inspired by another existing one, describe added value or differences | Fully: The manuscript implicitly addresses this by stating the goal was to integrate into existing programs, highlighting the value-add of XR rather than creating a new, separate intervention. | Introduction |
| 6 | Methods | Aims and objectives | M | Describe main objectives, predefined outcomes, KPIs, and how they will be measured | Fully: The objectives are clearly focused on implementation outcomes like feasibility, usability, and tolerability. | Abstract (Objective) |
| 7 | Methods | Blueprint summary | M | Describe design, key features of intervention, and implementation strategy/roadmap | Fully: Describes the implementation strategy of embedding XR directly into the existing clinical workflow of group sessions. | Introduction, Methods |
| 8 | Methods | Technical design | M | Describe reasons for developing/choosing tool, its functions, architecture, licensing, code availability, and fit within health enterprise architecture | Fully: Details the rationale for co-design, the offline nature of the apps, and the hardware used. | Methods (Co-Design) |
| 9 | Methods | Target | M | Define target (person, group, system, problem), site characteristics, and eligibility criteria | Fully: Defines the target population (adults with chronic pain), site characteristics, and eligibility criteria. | Methods (Participants) |
| 10 | Methods | Data | M | Describe data governance, ownership, protection, integration, legal framework, consent, cybersecurity, and hosting | Fully: Details data management, pseudonymization, storage on encrypted servers, and compliance with GDPR. | Methods (Data Management) |
| 11 | Methods | Interoperability | M | Describe interfaces, standards used (semantic, technical), and rationale | Partially: The manuscript implies a lack of interoperability by stating apps did not transmit data but doesn't explicitly discuss the rationale or standards considered. | Methods (Co-Design) |
| 12 | Methods | Participating entities | M | Describe implementing organizations, government involvement, partners, funders, and ownership of product/IP | Fully: Describes the implementing organization (BSA), partners (technical companies), and funders (Spanish Ministry of Health). | Throughout, Funding |
| 13 | Methods | Budget planning | M | Describe planned/actual budget, costs, duration covered, and budget for intervention | Partially: Identifies the funding source but does not provide details on the planned or actual budget for the implementation. | Funding |

| | | | | | | |
|---|---|---|---|---|---|---|
| 14 | Methods | Sustainability | M | Describe business/sustainability model, long-term strategies, institutionalization potential | Fully: Discusses sustainability through alignment with the national health strategy and institutional support from BSA. | Discussion (Implications) |
| 15 | Results | Coverage | M | Describe coverage level (international, national, regional), % of eligible population reached | Partially: Describes regional coverage (3 PCCs) but does not estimate the percentage of the eligible population reached. | Results (Sample characteristics ) |
| 16 | Results | Outcomes | M | Report actual outcomes using predefined measures | Fully: Reports on all predefined implementation and clinical outcomes with appropriate measures. | Results |
| 17 | Results | Lessons learned | M | Describe lessons learned, success factors, challenges, budget adherence, recommendations | Fully: The discussion is rich with lessons learned, including technical challenges (tablet sync) and user preferences (hand-tracking). | Discussion (Implications) |
| 18 | Results | Unintended consequences | NM | Describe unintended consequences (positive or negative) | Fully: Reports on transient discomfort (dizziness, fatigue) as unintended effects. | Results (Tolerability), Discussion |
| 19 | Discussion | Conclusion | M | Summary of conclusions and future implications | Fully: The conclusion summarizes the implementation findings and their future implications. | Conclusions |
| 20 | General | General | NM | Include regulatory approvals, registrations, COI, and relevant ethical considerations | Fully: Includes statements on regulatory classification (AEMPS), ethics approval, trial registration, and conflicts of interest. | Methods (Ethics), COI |

Adapted from: Perrin C, et al. iCHECK-DH: Guidelines and Checklist for the Reporting on Digital Health Implementations. J Med Internet Res. 2023;25:e46694. doi:10.2196/46694
